# Aging process and central auditory pathway: a study based on auditory brainstem evoked potential and frequency-following response

**DOI:** 10.31744/einstein_journal/2022AO6829

**Published:** 2022-05-18

**Authors:** Daniélli Rampelotto Tessele, Bruna Pias Peixe, Taissane Rodrigues Sanguebuche, Vitor Cantele Malavolta, Michele Vargas Garcia, Milaine Dominicini Sanfins

**Affiliations:** 1 Universidade Federal de Santa Maria Santa Maria RS Brazil Universidade Federal de Santa Maria, Santa Maria, RS, Brazil.; 2 Centro de Eletrofisiologia e Neuroaudiologia Avançada São Paulo SP Brazil Centro de Eletrofisiologia e Neuroaudiologia Avançada, São Paulo, SP, Brazil.

**Keywords:** Hearing, Evoked potentials, auditory, Hearing loss, Electrophysiology, Adult, Aging

## Abstract

**Objective:**

To analyze age-related changes in the central auditory pathway in healthy elderly individuals.

**Methods:**

A prospective, quantitative cross-sectional study. The caseload comprised 18 adults (mean age, 22.78 years) and 18 elderly individuals (mean age, 66.72 years) of both sexes, who met inclusion criteria. Subjects were submitted to basic audiological evaluation and related electrophysiologic tests: brainstem auditory evoked potential with click stimulus and frequency-following response.

**Results:**

Elderly individuals had higher wave and interpeak latencies (waves I, III and V and interpeaks I-V and III-V) of brainstem auditory evoked potential. Latencies of frequency following response waves A, E, F and O were also higher in elderly individuals. Frequency following response amplitudes were better in A than in D, F and O waves in these subjects. Likewise, interpeak intervals (V-A and V-O) were larger in elderly relative to adult individuals. Lower slope values were observed in elderly individuals.

**Conclusion:**

Brainstem auditory evoked potential and frequency-following response allowed appropriate assessment of age-related changes in the auditory pathway. Slower neural response to auditory stimuli suggests reduced synchrony between neural structures.

## INTRODUCTION

Population aging has been extensively discussed in the last few years. According to the World Health Organization (WHO), 1.2 billion people will be aged over 60 years in 2025.^( 1 )^ The aging process is associated with several losses, including presbycusis,^( 2 , 3 )^ which reflects the functional decline of structures of the peripheral auditory system.^( 1 )^

However, the most frequent complaint among the elderly is decline of speech understanding, particularly in noisy environments. This type of hearing impairment involves several central auditory structures and cannot be explained by increase in auditory thresholds alone.^( 4 )^

Two noninvasive objective potentials can be used to assess central auditory function: the brainstem auditory evoked potential (BAEP) with click stimulus, and frequency-following response (FFR). The BAEP is generated by structures located between the auditory portion of the vestibulocochlear nerve (cranial nerve VIII) and the brainstem and is therefore a short-latency potential.^( 5 )^

The FFR reflects the encoding of speech sounds along the central auditory nervous system,^( 6 )^ with subcortical responses^( 7 )^ and potential cortical contributions.^( 8 )^

The reliability of measures which involve behavioral assessment must be verified and validated. Difficulties to resolve and track dynamic sounds impair speech processing in elderly individuals.^( 9 )^ This study was designed to understand age-related FFR changes in this population.

Decreasing amplitudes and increasing latencies of BAEP and FFR waves are thought to be valid markers to assess the aging of the auditory system.

## OBJECTIVE

To analyze age-related changes in the central auditory pathway in healthy elderly individuals.

## METHODS

This prospective, quantitative cross-sectional study was examined and approved by the Research Ethics Committee of *Universidade Federal de Santa Maria* (UFSM), # 2.456.418, CAAE: 78740117.3.0000.5346. Procedures were carried out in the Speech Pathology and Auditory Electrophysiology Outpatient Service of a teaching hospital.

Individuals who agreed to participate signed an Informed Consent Form and a Confidentiality Form. Research procedures and related risks and benefits were detailed in the Informed Consent Form, in compliance with ethical standards of Resolution # 655/21 of the National Health Council.^( 10 )^

### Eligibility criteria

Subjects were recruited at the aforementioned Speech Pathology service. The following inclusion criteria were adopted: healthy individuals aged 19 to 76 years with normal auditory thresholds in both ears,^( 11 )^ no auditory complaints, bilateral type A tympanogram and right-handed according to the Edinburgh Handedness Inventory. Only elderly individuals with normal scores in the Mini Mental State Examination (MMSE) cognitive screening test were included.

Subjects with auditory complaints or obvious neurologic or psychiatric impairment were excluded.

### Caseload

This caseload comprised 36 healthy subjects: 18 adults aged 19 to 30 years (mean age, 22.78 years) and 18 elderly individuals aged 60 to 76 years (mean age, 66.72 years). Groups ( *i.e.* , adults and elderly individuals) were paired and comprised four males and 14 females each. The level of education was not examined.

### Sampling procedures

Subjects were submitted to hearing history-taking, meatoscopy, pure-tone threshold audiometry, speech audiometry, acoustic immitance testing, Edinburgh Handedness Inventory and MMSE.

### Research procedures

The BAEP test with click stimulus and the FFR test were carried out using Smart-EP equipment (Intelligent Hearing Systems, IHS). Tests were performed in a single day and lasted approximately 1 hour 30 minutes in both groups.

Individuals were accommodated in a reclining chair prior to skin cleaning with an abrasive paste. To ensure test reliability, tests were carried out with impedance values equal to or lower than 3kOhms and the number of artifacts did not exceed 10% of number of stimuli. Surface electrodes were attached using electrolyte paste and micropre tape. The active (Fz) and ground (Fpz) electrodes were placed on the central aspect of the front (upper and lower portion, respectively). Reference electrodes M1 and M2 were placed on the mastoid process of the temporal bone (left and right side). Stimulii were delivered using insert earphones.

**Figure 1:**
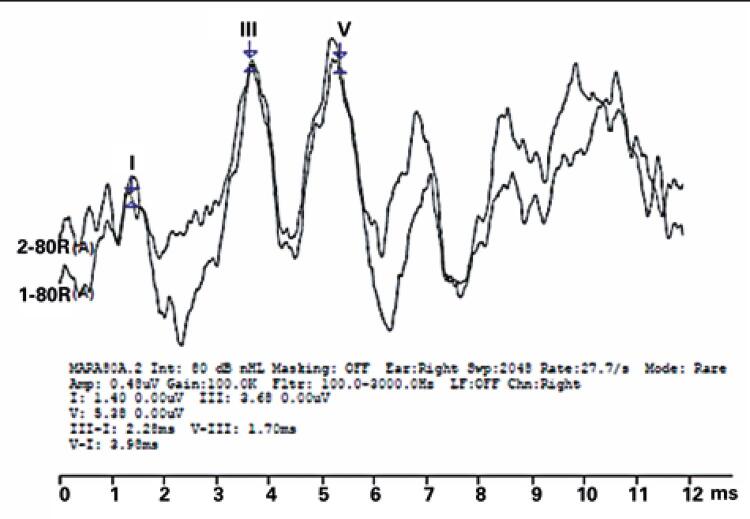
Brainstem auditory evoked potential with click stimulus - tracing recorded in one of research subjects

Click-evoked BAEP was recorded using the following settings: 80 dB nHL intensity, 12ms recording window, 2.048 sweeps, 27.7/s rate, 100Hz to 3.000Hz filter (high and low pass, respectively), 100.0K repetition rate, 100μs duration and rarefaction polarity. Waves I, III and V were marked in wave tracings ( Figure 1 ).

Click-evoked BAEP absolute latency was analyzed using Webster values.^( 12 )^Normality standards for a stimulation intensity of 80 dB nHL were as follows: wave I, 1.66 (Standard deviation, SD 0.10); wave III, 3.87 (SD 0.14); wave V, 5.68 (SD 0.11); interpeak I-III, 2.21 (SD 0.14); interpeak III-V, 1.81 (SD 0.10); interpeak I-V, 4.02 (SD 0.13).

In the FFR test (right ear only, *as per* guidelines), responses were elicited using the 40ms /da/ syllable provided by the manufacturer, with intensity of 80 dB nHL. Other settings were as follows: 60ms recording window, 40ms pre-stimulation, 100Hz to 3.000Hz filter (high and low pass, respectively), 125μs duration, 10.9/s rate and alterned polarity. A total of 3.000 sweeps were averaged and the resultant wave used in the analysis.^( 13 )^

**Figure 2:**
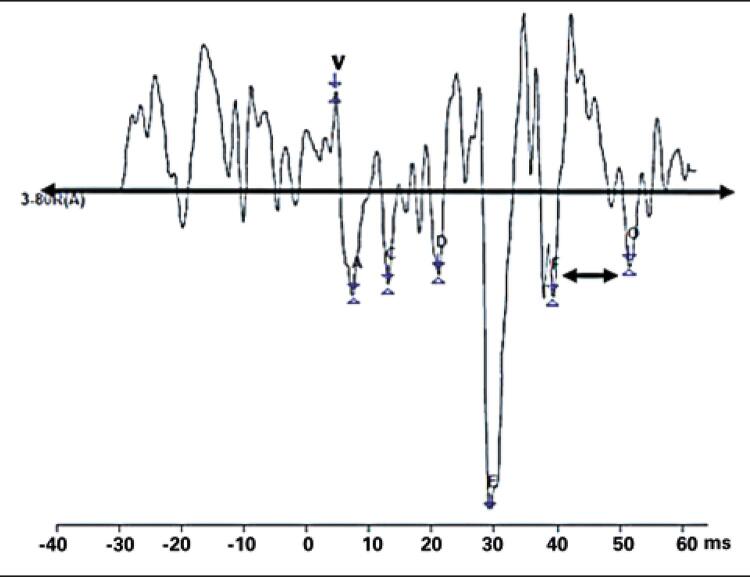
Frequency-following response - tracing recorded in one of research subjects

Whenever detected, waves V, A, C, D, E, F and O were marked in tracings ( Figure 2 ). The slope (amplitude V - amplitude A/latency A - latency V) was measured manually from baseline. Amplitudes were marked placing the cursor on the tip of each peak and troughs.

Absolute latency was estimated according to the following values Filippini et al:^( 13 )^ wave V, 6.46ms; wave A, 7.37ms; wave C, 18.32ms; wave D, 22.47ms; wave E, 30.64ms; wave F, 39.19ms; wave O, 48.01ms. Frequency analysis (pitch and harmonics) could not be carried out since the module required was not available at the organization.

Numerical variables were analyzed using descriptive statistics (mean and SD). Auditory potential data were compared using non-parametric tests (U or Mann-Whitney). The level of significance was set at 5% (<0.05).

## RESULTS

**Figure 3:**
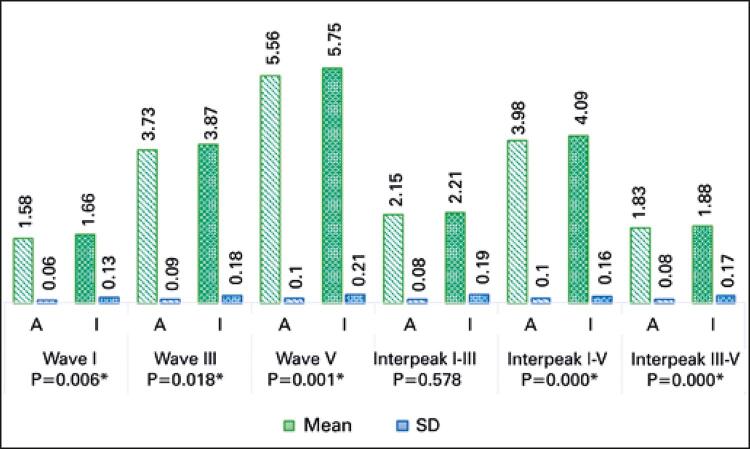
Comparison of latencies and interpeaks of brainstem auditory evoked potential with click stimulus (right ear)

**Figure 4:**
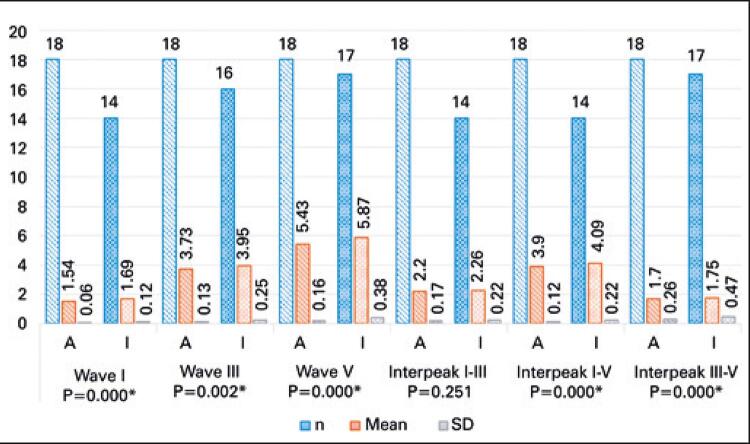
Comparison of latencies and interpeaks of brainstem auditory evoked potential with click stimulus (left ear)

Results shown in figures 3 and 4 represent absolute and interpeak latencies of waves I, III and V recorded in right and left ears. Different numbers (n) of adult and elderly subjects reflect absence of waves in testing.

* Statistically significant.

SD: standard deviation.

* Statistically significant.

SD: standard deviation.

**Figure 5:**
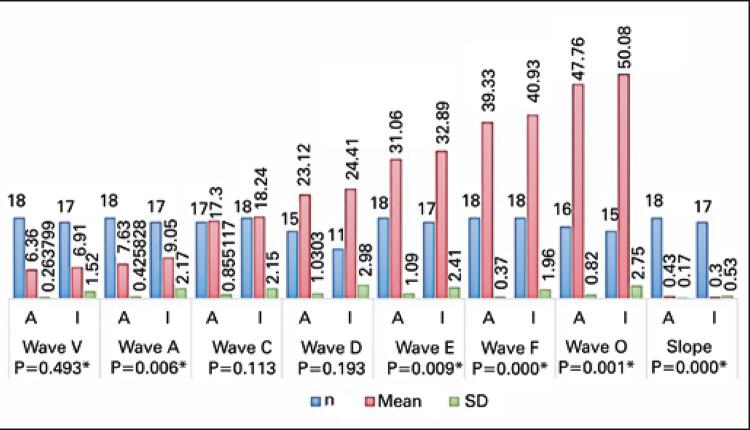
Comparison of latencies and slope of frequency-following response with speech stimulus between adult and elderly individuals

 Figures 5 to [Fig f06] 7 show the analysis of FFR test results (absolute latencies of waves, slopes, and amplitudes and interpeaks).

* Statistically significant.

SD: standard deviation.

**Figure 6 f06:**
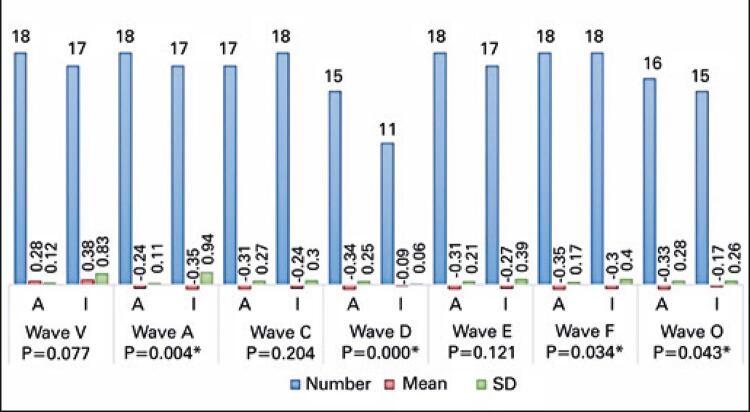
Comparison of amplitudes of frequency-following response with speech stimulus between adult and elderly individuals

* Statistically significant.

SD: standard deviation.

**Figure 7:**
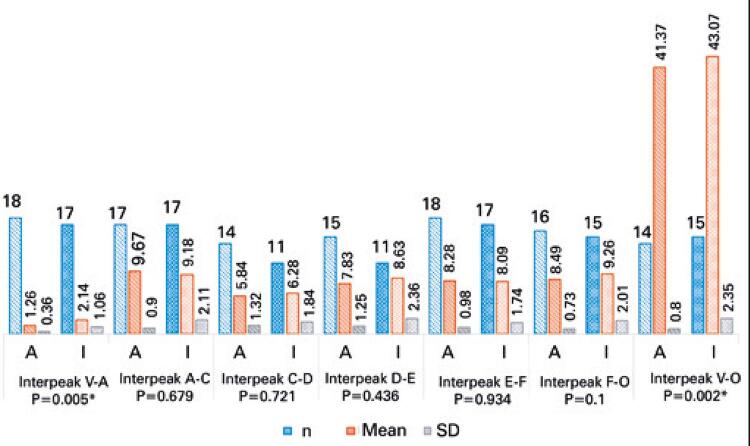
Comparison of interpeaks of frequency-following response with speech stimulus between adult and elderly individuals

* Statistically significant.

SD: standard deviation.

## DISCUSSION

This study provides insights into the aging of the auditory system in healthy subjects from the analysis of brainstem, subcortical and cortical auditory evoked potentials. Age-stratified investigation is vital for interpretation of diagnostic audiologic evaluation findings to support appropriate speech and hearing rehabilitation.

As hypothesized in this study, aging is associated with higher absolute latency of BAEP waves I, III and V, and with interpeak changes. These data show longer time is required for auditory stimuli to travel along the auditory pathway to the brainstem in elderly individuals and are consistent with prior studies^( 14 , 15 )^ addressing the impact of age on absolute BAEP latencies and interpeaks. According to Gupta et al.,^( 15 )^ central conduction involving the superior olivary complex and the inferior colliculus is compromised with increasing age.

Wave I was absent in the left ear in four out of 18 elderly individuals in this sample. These findings are in keeping with data reported by Matas et al.^( 16 )^ In that study, absence of BAEP waves was the most common change detected in subjects aged 70 to 79 years, indicating impaired neural synchrony.

As to FFR, most studies have reported on pediatric rather than elderly populations. Significant increase in latency and decrease in amplitude and slope values were observed in elderly individuals. Likewise, in a study^( 17 )^ with 34 subjects aged 22 to 77 years, aging was associated with longer wave latency and less robust responses with lower amplitudes.

Lower slope values in elderly individuals suggest deficient temporal synchrony in generators. Importantly, studies^( 18 , 19 )^ have shown that aging has a significant impact on temporal processing, interfering with the ability to track rapid changes in sound stimuli. Profant et al.^( 4 )^ also alluded to the significance of temporal processing for speech understanding in complex listening environments, as did other authors Skoe et al.,^( 20 )^who investigated 24 elderly individuals with normal auditory thresholds and reported stable FFR responses among adults and higher FFR latencies in elderly populations.

The auditory pathway is a sensory system that can be stimulated by environmental stimuli and communication. Hence, speech pathologists should submit patients to subjective and objective qualitative assessment. In this study, FFR was an effective tool for objective monitoring of auditory pathway aging. Different variables with potential impacts on the central auditory pathway of elderly individuals must be accounted for. These factors are also implicated in diagnosis and rehabilitation processes.

## CONCLUSION

Brainstem auditory evoked potential and frequency-following response allowed appropriate assessment of the aging of the auditory pathway in healthy elderly individuals. Slower neural response to auditory stimuli suggested reduced synchrony between neural structures.
